# The effect of intensive statin therapy in non-symptomatic intracranial arteries: The STAMINA-MRI sub-study

**DOI:** 10.3389/fneur.2023.1069502

**Published:** 2023-03-28

**Authors:** Jae Eun Sim, Ha-Na Song, Jong-Un Choi, Ji-Eun Lee, In Young Baek, Hwan-Ho Cho, Jong-Hoon Kim, Jong-Won Chung, Gyeong-Moon Kim, Hyun-Jin Park, Oh Young Bang, Woo-Keun Seo

**Affiliations:** ^1^Department of Neurology, Samsung Medical Center, Sungkyunkwan University School of Medicine, Seoul, Republic of Korea; ^2^Department of Electronic Electrical and Computer Engineering, Sungkyunkwan University, Suwon, Republic of Korea; ^3^School of Electronic and Electrical Engineering, Sungkyunkwan University, Suwon, Republic of Korea; ^4^Center for Neuroscience Imaging Research, Institute for Basic Science, Suwon, Republic of Korea

**Keywords:** artherosclerotic plaque, statin (HMG-CoA reductase inhibitor), Magnetic Resonace Imaging, systolic pressure, posterior cerebral arteries

## Abstract

**Background and aims:**

Pleiotropic effects of statins result in the stabilization of symptomatic intracranial arterial plaque. However, little is known about the effect of statins in non-symptomatic cerebral arteries. We hypothesized that intensive statin therapy could produce a change in the non-symptomatic cerebral arteries.

**Methods:**

This is a sub-study of a prospective observational study under the title of “Intensive Statin Treatment in Acute Ischemic Stroke Patients with Intracranial Atherosclerosis: a High-Resolution Magnetic Resonance Imaging (HR-MRI) study.” Patients with statin-naive acute ischemic stroke who had symptomatic intracranial artery stenosis (above 50%) were recruited for this study. HR-MRI was performed to assess the patients’ cerebral arterial status before and 6 months after the statin therapy. To demonstrate the effect of statins in the non-symptomatic segment of intracranial cerebral arteries, we excluded symptomatic segments from the data to be analyzed. We compared the morphological changes using cerebrovascular morphometry.

**Results:**

A total of 54 patients (mean age: 62.9 ± 14.4 years, 59.3% women) were included in this study. Intensive statin therapy produced significant morphological changes of overall cerebral arteries. Among the morphological features, the arterial luminal area showed the highest number of significant changes with a range from 5.7 and 6.7%. Systolic blood pressure (SBP) was an independent factor associated with relative changes in posterior circulation bed maximal diameter percentage change (beta −0.21, 95% confidence interval −0.36 to −0.07, *p* = 0.005).

**Conclusion:**

Intensive statin therapy produced a favorable morphological change in cerebral arteries of not only the target arterial segment but also non-symptomatic arterial segments. The change in cerebral arterial luminal diameter was influenced by the baseline SBP and was dependent on the topographic distribution of the cerebral arteries.

**Clinical Trial Registration**: ClinicalTrials.gov, identifier NCT02458755.

## Introduction

The apparent efficacy of statins in preventing cardiovascular events or stroke has imposed changes in clinical practice guidelines resulting in improved clinical outcomes (1, 2). Although the primary pharmacological effect of statins is the inhibition of cholesterol synthesis by blocking 3-hydroxy-3-methylglutaryl coenzyme A (HMG-CoA) reductase (3), statins also inhibit the production of isoprenoid intermediates exerting the so-called pleiotropic effect (4). This pleiotropic effect induces improvements in endothelial function (5), a reduction in inflammatory mediators, and an upregulation of endothelial nitric oxide synthase (6). All these effects are biologically associated with the stabilization and reduction in atherosclerotic plaques (7). Evidence from previous clinical trials also supports the pleiotropic effect–plaque regression link in patients with coronary artery disease or those with stroke and intracranial atherosclerosis (8–10).

Especially, we proved that high-dose statin therapy effectively reduced the stenosis degree, the volume of the enhanced plaque, and the wall area index of symptomatic intracranial atherosclerotic plaques in the article “Intensive Statin Treatment in Acute Ischemic Stroke Patients with Intracranial Atherosclerosis: High-Resolution Magnetic Resonance Imaging (STAMINA-MRI) study” (8). A meta-analysis supports the intensive statin effect on intracranial atherosclerosis (11).

However, the previous studies for intracranial atherosclerosis explored changes in the symptomatic target lesion (8, 10). The changes may be attributed not only to the plaque changes but also to the resolution of the thrombus on the ruptured plaque surface. Therefore, these results barely support the notion that intensive statin treatment stabilizes the surface or reduces the volume of non-symptomatic intracranial arterial atherosclerotic plaque. For the same reason, the clinical trials testing coronary atherosclerotic burden changes using intravascular ultrasound avoided target segments that have undergone angioplasty or more than 50% stenosis (9, 12). Therefore, we hypothesized that high-dose statin therapy could also produce a change in the non-symptomatic segment of intracranial cerebral arteries irrespective of other vascular risk factors.

Investigating intracranial arterial changes employed the grade of stenosis as the principal measure of the changes, and the manual segmentation of the target arterial segment is the standard method to assess the changes of the symptomatic intracranial arterial segment. However, to test all non-symptomatic intracranial arterial segments, manual segmentation is impossible and the automated quantification method is crucial. Therefore, we applied a novel tool to assess the global cerebral arterial morphological features using quantitative cerebral arterial morphometry which provides an automated assessment of cerebral arterial morphological quantification.

In this *post hoc* study of the STAMINA-MRI study, we explored the intensive statin effect on the morphological changes of non-symptomatic intracranial arteries.

## Materials and methods

### Study design

This is a sub-study of the STAMINA-MRI study, which is a single-arm, prospective, observational study, monitoring imaging and clinical outcomes of high-dose statins (atorvastatin 40–80 mg and rosuvastatin 20 mg) in symptomatic intracranial atherosclerosis. The details of the protocol were described elsewhere (8). In brief, the subjects were recruited between December 2011 and June 2017 from a university hospital. Patients with acute ischemic stroke within 7 days of the symptom onset, who had symptomatic intracranial artery stenosis (above 50%) at the proximal portion of the middle cerebral artery (MCA), basilar artery (BA), or the intracranial portion of the internal carotid artery, but not receiving statins were recruited for this study. Patients with the extracranial artery (carotid artery bifurcation and/or proximal vertebral artery) stenosis above 50%, stroke attributable to cardioembolic origin (atrial fibrillation, valvular heart disease, or aortic arch atheroma), and severe hepatic or renal dysfunction were excluded. All enrolled subjects received high-dose (atorvastatin 40–80 mg or rosuvastatin 20 mg) statin therapy for 6 months and were examined using a high-resolution (HR)-MRI before and 6 months after statin therapy. Blood samples were drawn on the days (initial and 6-month follow-up) of the HR-MRI examination after an overnight fast. To demonstrate the effect of statins in the non-symptomatic segment of intracranial cerebral arteries, we excluded symptomatic segments from the data to be analyzed.

The study protocol was approved by the institutional review board (SMC-IRB No. 2410-10-085). All patients provided signed informed consent prior to enrollment.

### HR-MRI

HR-MRI was performed using a 3-Tesla system (Achieva; Philips Medical System, Best, Netherlands) with a standard eight-channel head coil. The scan time of HR-MRI was approximately 40 min. This study used whole-brain three-dimensional (3D) MR angiography images with a time-of-flight protocol collected from each participant. With an isotropic voxel size configured to 0.284 × 0.284 mm^3^, the parameters were as follows: echo time, 4.59 ms; repetition time, 22 ms; flip angle, 23°; RBW, 130 Hz/pixel; GRAPPA factor, 3; and reference lines, 32.

### Quantitative cerebral arterial morphometry

**Figure 1:**
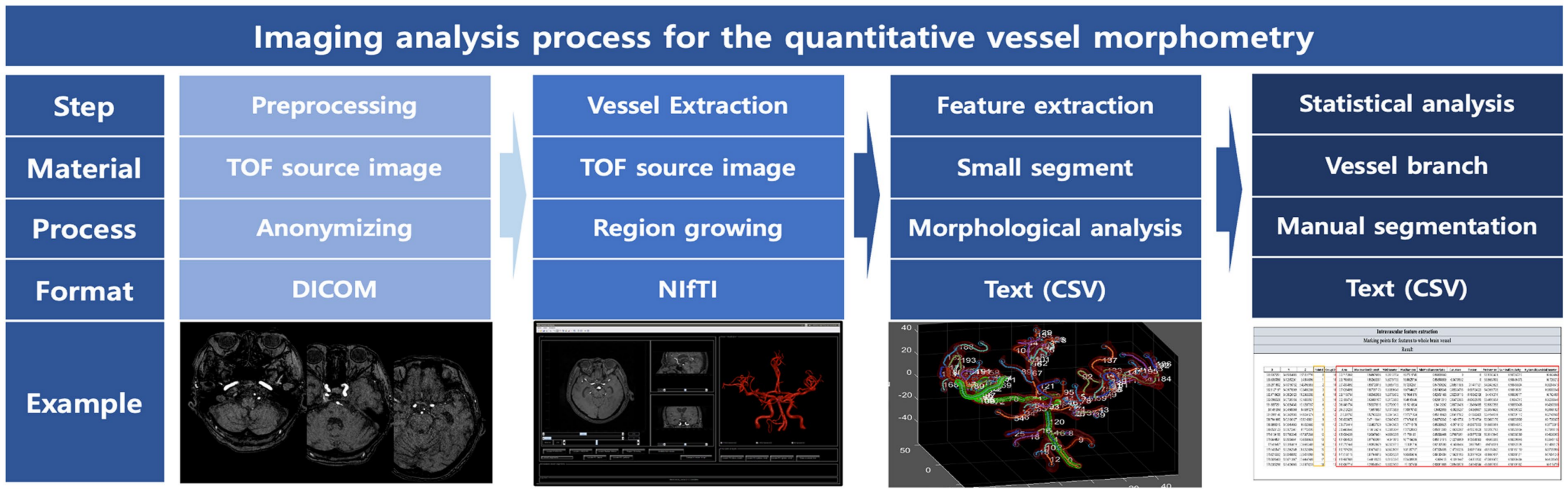
Overall process of the quantitative cerebrovascular morphometry. This tool was used by applying the open-source python library Vascular Modeling Toolkit (VMTK, http://www.vmtk.org), simple insight toolkit (SITK, https://sitk.org), and scikit-image (skimage, https://scikit-image.org).

The morphological features of cerebral arteries were extracted through a series of processes named quantitative cerebral arterial morphometry (composed of preprocessing), vessel and feature extraction, and post-analytic process. Every step was performed using in-house software (Figure 1). First, after the conversion of a two-dimensional DICOM file of MR angiography into the Neuroimaging Informatics Technology Initiative format, we extracted a 3D contour of cerebral arteries using a region-growing method. Thereafter, cerebral arterial morphological features were extracted by calculating the centerline with a “vascular modeling toolkit.” To specify feature modeling, the dissection of the iso-surfaces for vessel surface model generation initially occurs. A continuous 3D space divides into a myriad of cells uniformly based on respective vertices of iso-surfaces. Thereafter, major arterial centerlines from the boundary surface of each cell were extracted. The process provides a bundle of fine-arterial segments (0.28-mm distance between neighboring segments) with their morphological features including luminal area, maximal inscribed sphere radius, minimal luminal diameter, maximal luminal diameter, minimal–maximal diameter ratio, curvature, torsion, perimeter, and luminal circularity (Supplementary Table S1). Finally, the extracted arterial segments were allocated into arterial territories (74 arterial branches). These branches were grouped into 19 groups (chunks; Supplementary Table S2) based on the regional and functional similarities. To compare the difference of each feature between the initial value and that at 6 months, the mean values of the features for each chunk were calculated. The magnitude of changes was presented as % changes: [(Mean_initial_ – Mean_6months_)/ Mean_initial_] × 100 (%).

### Statistical analysis

Categorical variables were summarized according to frequencies and percentages, whereas continuous variables were expressed as mean ± standard deviation. The intra-individual comparisons for the continuous variables were performed using the Wilcoxon-signed rank test. The independent association between the vascular risk factors with the changes in arterial morphological features was tested using multivariable linear regression analyses. In all the analyses, a *value of p* of *<*0.05 was considered to be statistically significant. R software (R version 4.1.0, The R Foundation for Statistical Computing) and Python software (Python version 3.8.18) were used for all statistical analyses.

## Results

Among the 77 subjects in the STAMINA-MRI study, 54 subjects (mean age: 62.9 ± 14.4 years, 59.3% women) whose cerebral arterial features were successfully extracted were analyzed (Supplementary Figure S1). The mean number of analyzed vessels is 37.2 ± 9.6. The details of the baseline characteristics of patients including vascular risk factors, laboratory findings, and time interval for the MRI are summarized in Table 1. The leading vascular territory of the index infarct was the right MCA territory (46.3%), followed by the left MCA territory (40.7%) and BA territory (13.0%). Atorvastatin and rosuvastatin were prescribed for 15 (27.8%) and 39 (72.2%) subjects, respectively. No subject was prescribed an additional lipid-lowering regimen such as ezetimibe or proprotein convertase subtilisin/kexin type 9 inhibitor. This intensive statin therapy significantly reduced total cholesterol, low-density lipoprotein (LDL) cholesterol, and triglycerides by 35.0, 50.8, and 25.4%, respectively (Table 1). The median time interval between the initial HR-MRI evaluation and follow-up HR-MRI was 179.43 ± 19.39 days.

**Table 1:** Baseline characteristics, vascular risk factors, and laboratory findings of the study patients.

Clinical parameters (*n* = 54)	
Age, year	62.9 ± 14.4
Female, *n* (%)	32 (59.3)
Systolic blood pressure, mmHg	147.9 ± 21.6
Diastolic blood pressure, mmHg	83.2 ± 13.1
Body mass index (kg/ m^2^)	24.6 ± 3.9
Hypertension, *n* (%)	32 (59.3)
Diabetes mellitus, *n* (%)	20 (37.0)
Hyperlipidemia, *n* (%)	24 (44.5)
Atrial fibrillation, *n* (%)	0 (0)
Current smoking, *n* (%)	11 (20.4)
Current alcohol consumption, *n* (%)	16 (29.6)
Previous stroke, *n* (%)	9 (16.7)
Imaging acquisition	
Onset to initial MR time (day)	3.9 ± 2.0
Onset to follow up MR time (day)	179.4 ± 19.4
Vascular territory of infarction	
Middle cerebral artery, right	25 (46.3)
Middle cerebral artery, left	22 (40.7)
Basilar artery	7 (13.0)
NIHSS score	2.9 ± 4.0
*Statin*	
Atorvastatin 40 mg or 80 mg, *n* (%)	15 (27.8)
Rosuvastatin 20 mg, *n* (%)	39 (72.2)
*Lipid profile*	
Initial total cholesterol, mg/dL	186.5 ± 43.6
Initial triglycerides, mg/dL	156.1 ± 96.0
Initial LDL-cholesterol, mg/dL	117.6 ± 35.9
Initial HDL-cholesterol, mg/dL	49.4 ± 15.5
Follow-up total cholesterol, mg/dL	121.3 ± 23.8
Follow-up triglycerides, mg/dL	116.4 ± 51.7
Follow-up LDL-cholesterol, mg/dL	58.3 ± 18.7
Follow-up HDL-cholesterol, mg/dL	50.8 ± 15.0

HDL, high-density lipoprotein; LDL, low-density lipoprotein; NIHSS, National Institute Health Stroke Scale.

### Changes in the arterial morphological features

The morphological features of symptomatic atherosclerotic plaques measured at the most stenotic segment showed similar trends as those in the mother study (STAMINA-MRI). The stenosis grade (15.2%), remodeling index (5.3%), and wall area index (18.6%) improved after 6 months of intensive statin therapy in this sub-study (Table 2).

**Table 2:** Changes of the vessel wall features after 6 months of high-dose statin therapy.

Features	Initial	Follow-up	*p* ^*^
*Symptomatic artery*			
Stenosis grade (%)	75.7 ± 20.1	64.2 ± 21.1	<0.01
Remodeling index	1.1 ± 0.3	1.1 ± 0.2	0.04
Wall area index	7.2 ± 4.4	5.9 ± 3.5	<0.01
*Non-symptomatic artery*			
Area, mm^2^	5.76	5.92	<0.01
Maximum inscribed sphere radius, mm	1.00	1.01	<0.01
Minimal diameter, mm	2.06	2.08	<0.01
Maximal diameter, mm	2.91	2.95	<0.01
Min-max diameter ratio	0.78	0.78	<0.01
Curvature	0.12	0.12	0.64
Perimeter, mm	8.45	8.56	<0.01
Luminal circularity	0.95	0.94	0.69

HDL, high-density lipoprotein; LDL, low-density lipoprotein. ^*^The Wilcoxon-signed rank tests were performed.

**Figure 2:**
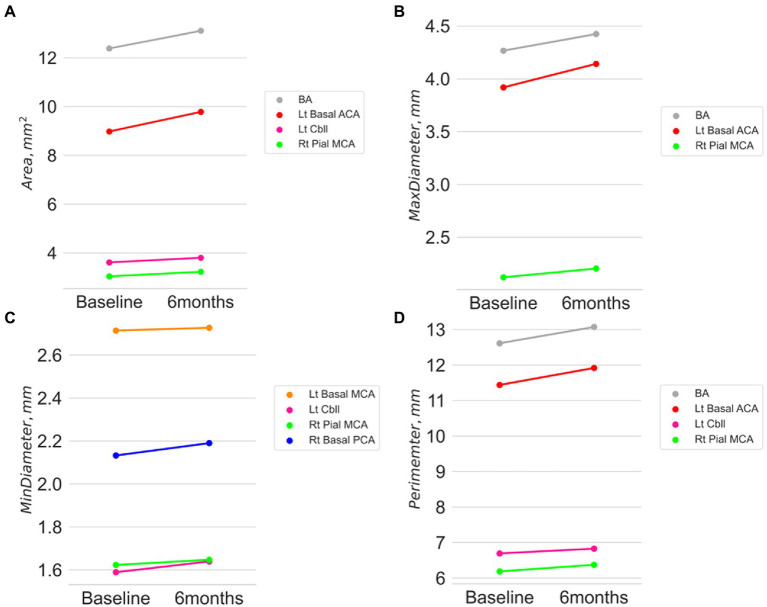
Absolute changes of arterial luminal area **(A)**, the maximum diameter **(B)**, the mini diameter **(C)**, and the perimeter **(D)** after 6 months of intensive statin therapy.

**Figure 3:**
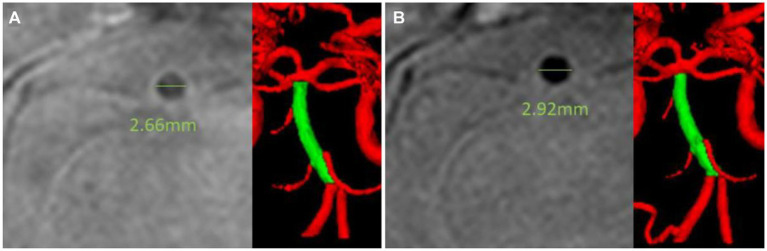
Illustrative case showing intracranial arterial lumen diameter changes. A 67-year-old man presented with right middle cerebral arterial territorial infarction. Intensive statin therapy with rosuvastatin 20 mg for 6 months reduced LDL cholesterol level from 164 mg/dl to 46 mg/dl. A high-resolution vessel wall image revealed that the luminal diameter of his basilar artery increased from 2.66 mm **(A)** to 2.92 mm, **(B)** although there was no evidence of plaques on the basilar artery segment. The mean luminal maximal diameter of his basilar artery measured by the cerebrovascular morphometry increased by 9.7%.

In contrast, the morphological changes of non-symptomatic arteries in this study were assessed for the entire cerebral arterial tree after the exclusion of the symptomatic stenosis segment. A 6-month statin therapy increased arterial luminal diameter and its derivatives except for the curvature and luminal circularity (Table 2). The cells with significant changes are spread over the anterior and posterior circulation and in large basal and pial small arteries. Among the morphological features, the arterial luminal area showed the highest number of significant changes after the intensive statin therapy with a range from 5.7 to 6.7% according to the arterial segments with statistical significance (Supplementary Table S3). The maximal diameter showed an increasing trend with a range from 3.3 to 4.4%. Other features reflecting arterial luminal size, such as inscribed sphere radius, minimal diameter, or perimeter, also increased. In contrast, curvature, maximal-to-minimal radius ratio, and luminal circularity showed no significant changes after the intensive statin therapy. No apparent trend of association with the morphological changes appeared in terms of the distribution of arterial segments (anterior circulation versus posterior circulation; proximal basal cerebral arteries versus small pial arteries). Figure 2 shows a significant increase in the cerebral arterial luminal area (mm^3^) (BA 12.38 ± 3.14 vs. 13.10 ± 3.26; *p =* 0.014, Lt basal ACA 8.98 ± 3.69 vs. 9.78 ± 4.85; *p =* 0.028, Rt pial MCA 3.04 ± 1.30 vs. 3.23 ± 1.42; *p =* 0.006, Lt cbll 3.61 ± 2.05 vs. 3.80 ± 0.12; *p =* 0.018), the maximal arterial diameter (mm) (BA 4.27 ± 0.64 vs. 4.42 ± 0.68; *p =* 0.009, Lt basal ACA 3.92 ± 1.07 vs. 4.14 ± 1.22; *p =* 0.011, Rt pial MCA 2.12 ± 0.42 vs. 2.20 ± 0.52; *p =* 0.006), the minimal arterial diameter (mm) (Lt basal MCA 2.71 ± 0.77 vs. 2.73 ± 0.51; *p =* 0.048, Rt basal PCA 2.13 ± 0.52 vs. 2.19 ± 0.53; *p =* 0.013, Rt pial MCA 1.62 ± 0.24 vs. 1.65 ± 0.20; *p =* 0.038, Lt cbll 1.59 ± 0.24 vs. 1.64 ± 0.25; *p =* 0.026), and the perimeter (mm) (BA 12.61 ± 1.88 vs. 13.07 ± 2.17; *p =* 0.047, Lt basal ACA 11.44 ± 3.46 vs. 11.92 ± 3.64; *p =* 0.015, Rt pial MCA 6.18 ± 1.38 vs. 6.37 ± 1.34; *p =* 0.006, Lt cbll 6.69 ± 2.62 vs. 6.83 ± 1.90; *p =* 0.024). Figure 3 demonstrates an example case of luminal diameter changes (9.7% increase) in the non-symptomatic BA segment.

### Factors affecting the vascular morphological feature changes

**Figure 4:**
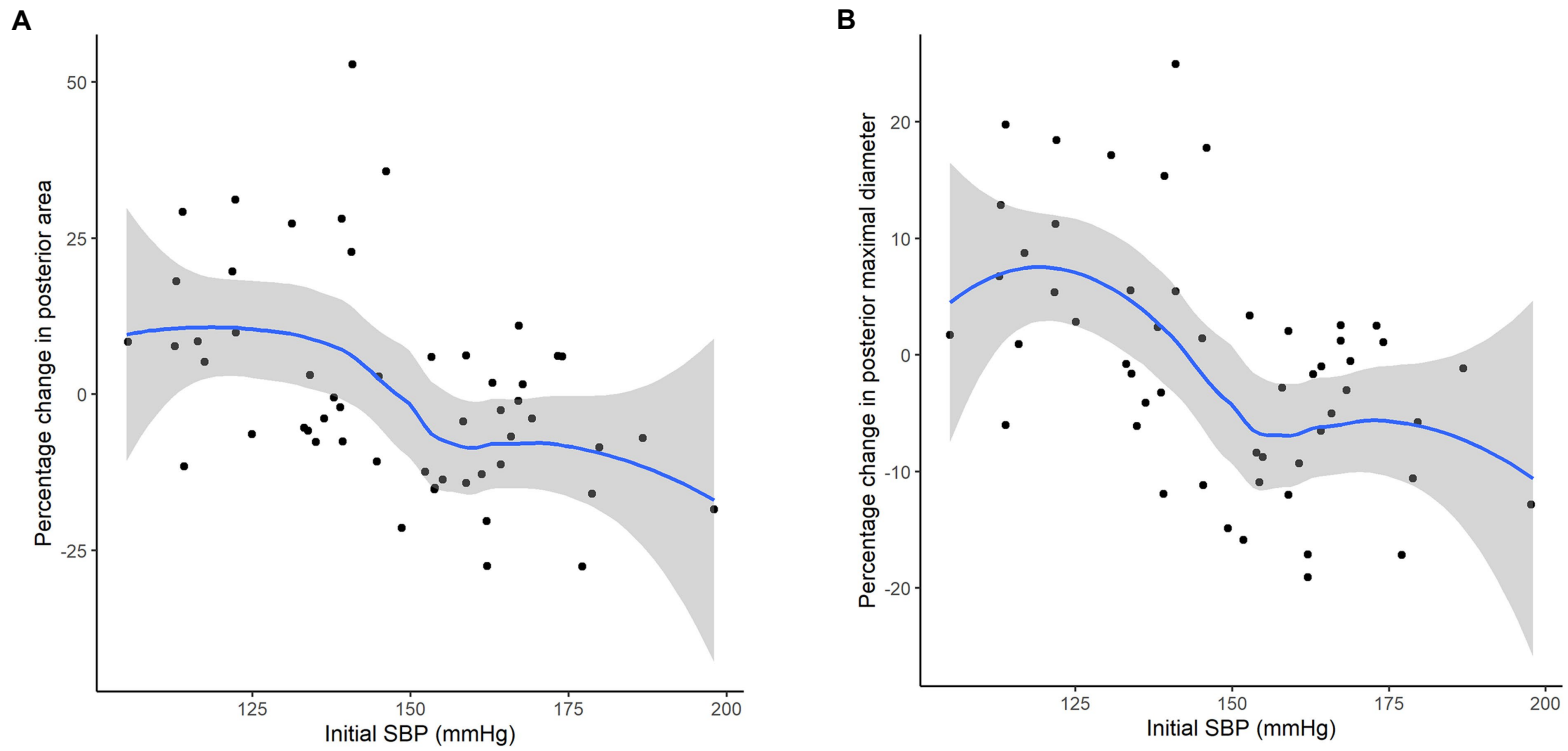
Correlation between initial SBP on the percent changes of arterial luminal area **(A)** and the maximum diameter **(B)**. The plots were generated by the approximated polygonal curve approximation for the correlation.

The interfering effect of demographics and the vascular risk factors on the changes in arterial morphological features is presented on a heatmap (Supplementary Figure S2). Systolic blood pressure (SBP) and diastolic blood pressure were negatively associated with the changes of parameters of the arterial luminal size such as the area, maximal diameter, or perimeter exclusively in the posterior circulation bed. A sensitivity analysis performed using a subset of stenosis-free cerebral arteries showed similar trends. This trend was reproduced in an adjusted linear regression using posterior circulation bed maximal diameter difference: SBP was an independent factor associated with relative changes in posterior circulation bed maximal diameter % change (beta −0.21, 95% confidence interval − 0.36−−0.07, *p =* 0.005) (Table 3; Figure 4).

**Table 3:** Clinical and laboratory features affecting changes in the maximal diameter in posterior circulation bed branches.

	Univariable analysis		Multivariable analysis	
	β (95% CI)	*p*	β (95% CI)	*p*
Age	−0.17 (−0.98–0.46)	0.12	−0.07 (−0.28–0.14)	0.50
Sex	5.56 (−0.48–11.59)	0.07	0.91 (−5.43–7.26)	0.77
Body mass index (kg/m2)	−0.12 (−0.94–0.69)	0.77		
Hypertension	−1.41 (−7.63–4.81)	0.65		
Diabetes	1.19 (−4.37–8.16)	0.55		
Coronary vascular disease	4.50 (−3.19–12.19)	0.25		
Current smoking	1.06 (−2.79–4.90)	0.58		
LDL-C change (%)	−0.06 (−0.20–0.07)	0.35		
Statin therapy duration (days)	−0.10 (−0.25–0.06)	0.21		
Cilostazol use	3.28 (−9.06–15.61)	0.60		
Systolic blood pressure	−0.23 (−0.36 to −0.11)	<0.01	−0.21 (−0.36 to −0.07)	<0.01

LDL, low-density lipoprotein.

## Discussion

This study demonstrated that intensive statin therapy produced favorable changes in the intracranial morphological features of not only the symptomatic atherosclerotic segment but also of the normal-appearing segments in stroke patients with intracranial artery stenosis. These intracranial arterial luminal changes of non-symptomatic segments were independent of the magnitude of LDL reduction and were not correlated with the changes in symptomatic arterial features suggesting the presence of a distinct mechanism. Instead, baseline SBP negatively interfered with the arterial luminal size metrics exclusively in the posterior circulation arterial bed.

Published literature shows that intensive statin therapy leads to atherosclerotic plaque regression in patients with coronary artery disease, and the effect is apparent in both acute and non-acute coronary syndrome (13). In patients with stroke, the patterns were construed similarly to those with coronary artery disease but with limited evidence. The STAMINA-MRI study provides evidence that intensive statin therapy stabilizes symptomatic intracranial atherosclerotic plaques (8). In addition to the STAMINA-MRI results, this study expands the substrate under the influence of intensive statin therapy to non-diseased arterial segments and renews a debate on the pleiotropic effects of statin.

It should be noted that intracranial arteries have features distinct from coronary artery bed regarding their histology and pathology. The pathogenetic mechanisms of intracranial arteries are complex and heterogeneous including atherosclerosis, arterial dissection, moyamoya disease, or vasculitis (14). The clinical benefits of statins are attributable mainly to reducing LDL cholesterol that is a key player driving the lipid component of atherogenesis (15). The increased luminal diameter or area in this study reflects the plaque regression effect by the marked LDL cholesterol lowering coupled with the increased HDL cholesterol, called the reverse cholesterol transport (16). However, this mechanism is insufficient to explain the phenomenon observed in this study because the increased luminal size metrics were consistent even in the arterial segments without stenosis (Supplementary Table S3). Rather, the pleiotropic effect, i.e., endothelial cell-mediated vasodilatation, vascular smooth muscle cell-related vascular wall remodeling, angiogenesis to facilitate endothelial progenitor cell increment (17), or anti-inflammatory response in monocytes or macrophages (4), is acceptable in explaining the non-stenotic intracranial arterial changes in reaction to statin therapy. The lack of association between LDL cholesterol reduction and the change in the arterial luminal size strongly supports this concept. An association between the parameters of symptomatic and non-symptomatic arterial segments was not found (Supplementary Table S4). Therefore, it can be seen that the change in the symptomatic and non-symptomatic arterial segments occurred through different mechanisms.

This study is unveiling the unsolved phenomenon of the statin effect in acute stroke patients. A number of studies have consistently reported the effect of statins on improved functional outcomes and decreased mortality in patients with non-atherosclerotic stroke in a dose-dependent manner. However, statin therapy failed to show a reduction in recurrent stroke risk (18). Observations in cardioembolic stroke, which are exclusive to atherosclerotic stroke, confirmed this association (19, 20). The increased cerebral arterial luminal metrics in this study can be extrapolated as improved cerebral perfusion and also be a clue for the unexpected link between statin therapy and stroke outcomes. These pleiotropic effects of statin offer a reason for the prevention and treatment of ischemic stroke.

We should also note the fact that SBP exerts a strong influence on the arterial luminal changes exclusively in the posterior circulation bed. Among the vascular risk factors, hypertension and dyslipidemia were the dominating determinants for vascular aging (21). All participants in this study were receiving intensive statin therapy with resultant large LDL reduction. Therefore, only blood pressure could regulate the progression of vascular aging represented by the diameter metrics of the cerebral arteries. The regional difference in terms of the SBP effect on arterial morphology is an interesting finding. Histologically, cerebral arteries of the posterior circulation bed had larger internal elastic lamina, less elastin, more concentric intimal thickening, and decreased collagen, and these histologic features are known to be vulnerable to vascular aging (22). Therefore, we presumed that the higher vulnerability to the progression of vascular aging in the posterior circulation bed was associated with the topographic disparity of the statin effect. Chronic hypertension disrupts the vasoconstrictor effect of sympathetic activation, so-called protective in response to hypertension (23). The relatively poor sympathetic innervation of the posterior cerebral arteries indeed is another possible cause of the distinguished vulnerability to hypertension. Thus, chronic hypertension induced posterior cerebral artery stiffness by hypertrophy and inward remodeling of cerebral arteries and arterioles (24). Therefore, the high vulnerability of the posterior cerebral arteries to the pro-aging effect of hypertension might be attributed to the less active protective effect of intensive statin therapy in posterior cerebral arteries.

It should be noted that we introduced cerebral arterial morphometry as a new tool for the quantitative assessment of cerebral arterial morphological features. It is hard to deny that the complexity and the inter-individual variability of the cerebral vasculature limited the quantitative evaluation of the cerebral arteries, and, until now, manually assessed semiquantitative analyses were the mainstay of the cerebrovascular morphological evaluation. Recently, a method quantifying cerebral arterial diameter using MR angiography was reported (25). However, this study has provided a vessel diameter in two-dimensional space only. In this study, we propose a new technique, the so-called quantitative cerebrovascular morphometry. The quantitative cerebrovascular morphological features are ready to be used for future studies such as the assessment of therapeutic effect, tracking chronological changes over time, or a comparison between different groups.

This study has several important limitations. First, this was a single-center study with a small number of patients comprising only the Korean population. The sample size of the study may have not been sufficient to detect a differential effect of high-dose statins in the localization and size of non-symptomatic arteries. Therefore, the generalizability of the study findings may be limited and needs to be validated in a larger study including a multiethnic population. Second, although this was a prospective study, there was significant variability in the time interval between initial and follow-up HR-MRIs. The variability in statin treatment duration may have also affected the results. Third, quantitative cerebrovascular morphometry used in this study is inappropriate to evaluate the arterial segments with signal loss, and the morphological changes were assessed by time-of-flight images that are vulnerable to image distortion. However, in this study, non-symptomatic arterial segments which are relatively less vulnerable to artifacts were the target of the analysis. Furthermore, the features employed in this study were not the measurement of a single point but the mean of spots comprising a specific branch. Therefore, the influence of image distortion was expected to be minimized. Lastly, in addition to statin, other drugs can exert a positive effect on cerebral arteries. Especially, cilostazol, a phosphodiesterase inhibitor, is a well-known drug with a vasodilatory effect. Therefore, we also confirmed the effect of cilostazol which affects endothelial cell function. However, no interaction was found between cilostazol and the effect of intensive statin therapy (Supplementary Figure S2).

## Conclusion

High-dose statin therapy increased the cerebral arterial diameter irrespective of atherosclerotic stenosis providing a clue for the non-plaque stabilizing beneficial statin effect in patients with acute stroke. The change in cerebral arterial luminal diameter was influenced by the baseline SBP and was dependent on the topographic distribution of the cerebral arteries. In conclusion, cerebrovascular morphometry was a useful tool in investigating the morphological changes in cerebral arteries in a quantitative manner, and the pleiotropic effects of statin improved non-symptomatic cerebral arteries.

## Data availability statement

The original contributions presented in the study are included in the article/Supplementary material, further inquiries can be directed to the corresponding authors.

## Ethics statement

The study protocol was approved by the institutional review board (SMC-IRB No. 2410-10-085). All patients provided signed informed consent prior to enrollment. The patients/participants provided their written informed consent to participate in this study.

## Author contributions

JS established the study concept, analyzed and interpreted the data, and wrote the manuscript. J-WC established the study database and made a critical revision of the manuscript. W-KS and OB established the study idea, database, and made critical revisions to the manuscript with intellectual contents. The statistical analysis was conducted by J-WC and JS. All authors contributed to the article and approved the submitted version.

## Funding

The study was supported in part by funds from Samsung Medical Center (OTX000036: OB), Dong-A Pharma, Inc. (PHO112519; OB), and the National Research Foundation of the Republic of Korea (NRF-2020M3E5D2A01084715; W-KS). The funders had no role in the study design, data collection and analysis, decision to publish, or manuscript preparation.

## Conflict of interest

The authors declare that the research was conducted in the absence of any commercial or financial relationships that could be construed as a potential conflict of interest.

## Publisher’s note

All claims expressed in this article are solely those of the authors and do not necessarily represent those of their affiliated organizations, or those of the publisher, the editors and the reviewers. Any product that may be evaluated in this article, or claim that may be made by its manufacturer, is not guaranteed or endorsed by the publisher.
