# Supplementary material for: The effect of intensive statin therapy in non-symptomatic intracranial arteries: The STAMINA-MRI sub-study
Source: Front Neurol. 2023 Mar 28;14:1069502. doi: 10.3389/fneur.2023.1069502 (PMC10088516; doi:10.3389/fneur.2023.1069502)
Supplement: Supplementary file 1 [file Data_Sheet_1.PDF]

Figure S1. Patients enroll tree.

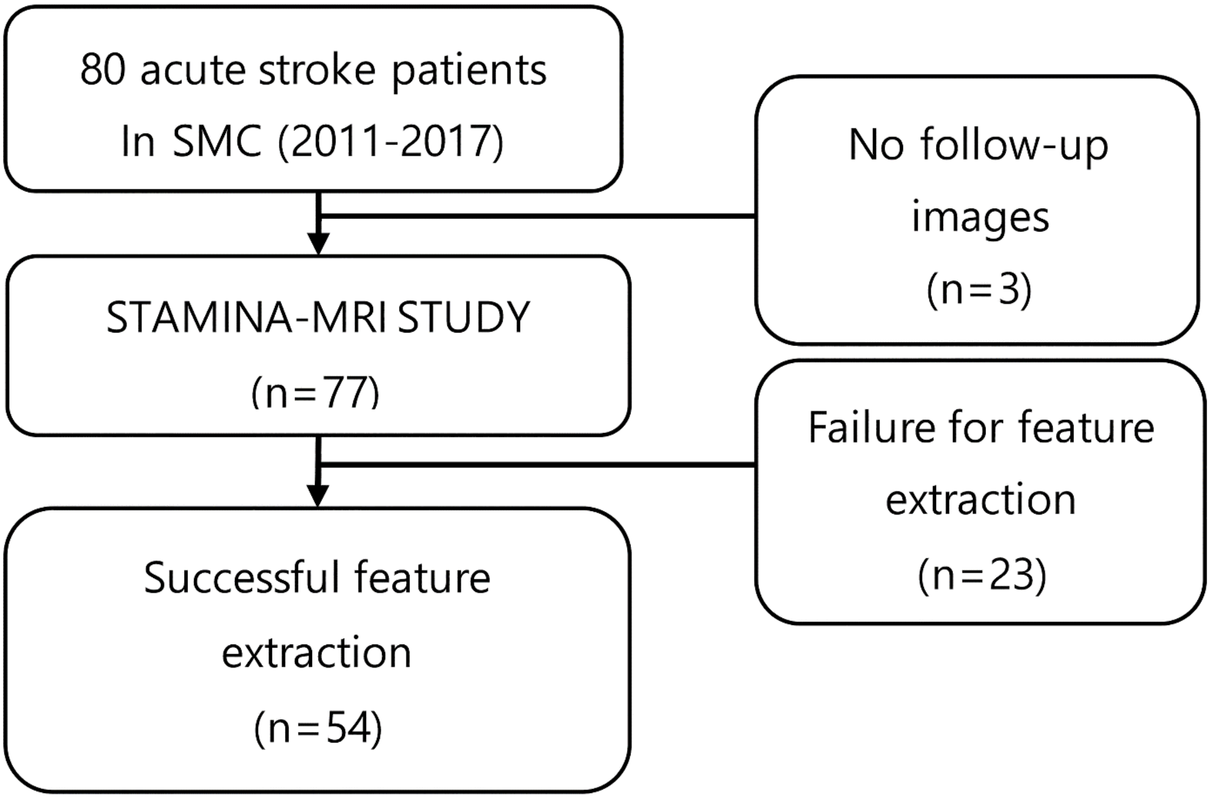

Figure S2. The differential association between baseline characteristics and the morphological features of cerebral arteries according to the location and the size of cerebral arteries.

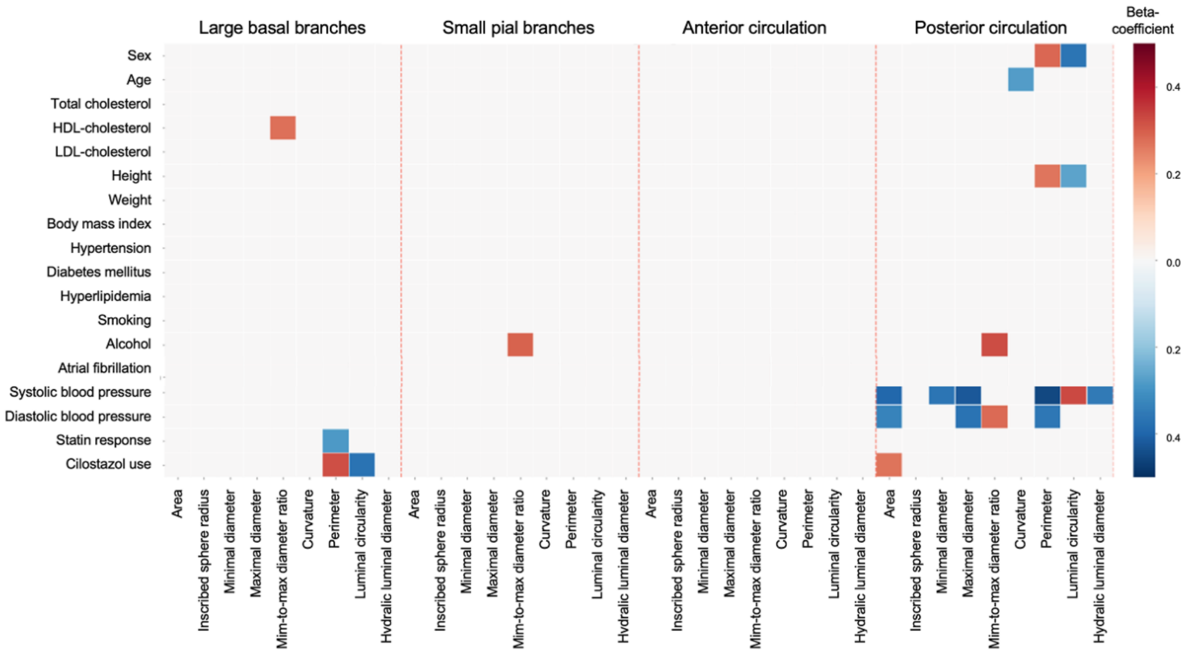

The colored cells mean that there was a significant ($P<0.05$) correlation between clinical characteristics (raw) and the relative changes of each vessel feature between the initial and 6 months after (column). The association was tested by Pearson correlation test.

The color bar represents the magnitude of the relative change (% change) of the feature between the initial and 6 months after.

Table S1. The explanation of features

X,Y, Z	Center line coordinates
Area	Cross-sectional area at point location
Max inscribed sphere radius	Maximal radius of inscribed sphere from point to vessel wall
Mini diameter	Minimal diameter of vessel cross-section at point
Max diameter	Maximal diameter of vessel cross-section at point
Min-max diameter ratio	Mini diameter / Max diameter
Curvature	Curvature of vessel centerline at point
Torsion	Torsion of vessel centerline at point
Hyduralic luminal diameter	Diameter defined by a new formula in the existing literature
Peremeter	Cross-sectional perimeter at point
Luminal circularity	Shape feature that indicates how close the cross-section is to a circle

Table S2. The Arterial Territories Used in This Study

Rt ICA	rt internal carotid a., rt ophthalmic a.
Lt ICA	lt internal carotid a., lt ophthalmic a.
Rt Basal MCA	rt MCA m1, rt m2 superior, rt m2 inferior
Lt Basal MCA	lt anterior basal MCA, lt MCA superior, lt MCA inferior
Rt Basal ACA	rt ACA a1,a2
Lt Basal ACA	lt ACA a1,a2
Rt Pial MCA	rt orbitofrontal a., rt MCA prerolandic a., rt MCA rolandic a., rt MCA anterior parietal a., rt MCA posterior parietal a., rt MCA angular a., rt MCA posterior temporal a., rt MCA midtemporal a., rt MCA anterior temporal a.
Lt Pial MCA	lt MCA orbitofrontal a., lt MCA prerolandic a., lt MCA rolandic a., lt MCA anterior parietal a., lt MCA posterior parietal a., lt MCA angular a., lt MCA posterior temporal a., lt MCA midtemporal a., lt MCA anterior temporal a.
Rt Pial ACA	rt ACA orbitofrontal a., rt ACA frontopolar a., rt ACA callosomarginal a., rt ACA pericallosal a.
Lt Pial ACA	lt ACA orbitofrontal a., lt ACA frontopolar a., lt ACA callosomarginal a., lt ACA pericallosal a.
Rt VA	rt vertebral a.
Lt VA	lt vertebral a.
Rt Basal PCA	rt PCA p1, p2, p3
Lt Basal PCA	lt PCA p1, p2, p3
Rt Pial PCA	rt posterior communicating a., rt hippocampal a., rt PCA anterior temporal a., rt PCA posterior temporal a., rt parieto-occipital a., rt calcarine a.
Lt Pial PCA	lt posterior communicating a., lt hippocampal a., lt PCA anterotemporal a., lt PCA posterior rt temporal, lt parieto-occipital a., lt calcarine a.
Rt Cbll	rt posterior inferior cerebellar a., rt anterior inferior cerebellar a., rt superior cerebellar a.
Lt Cbll	lt posterior inferior cerebellar a., lt anterior inferior cerebellar a., lt superior cerebellar a.
BA	basilar a.

Tabel S3. Relative Changes of the Morphological Features According to the Distribution of Cerebral Arteries

Group	Area		Insc. Sphere R		Min. diameter		Max. diameter		Min-Max ratio		Curvature		Perimeter		Circularity	
	%*	*P*†	%*	*P*†	%*	*P*†	%*	*P*†	%*	*P*†	%*	*P*†	%*	*P*†	%*	*P*†
BA	5	0.03	1.8	0.12	1.4	0.24	3.3	0.02	-0.7	0.24	-0.9	0.86	3.2	0.09	0	0.82
Lt ACA basal	7.5	0.06	1.8	0.22	2.8	0.11	4.4	0.04	-0.3	0.30	1.4	0.34	2.9	0.04	-0.1	0.42
Lt ACA pial	4.8	0.97	-0.9	0.42	-1.3	0.28	3.7	0.66	-2.4	0.12	-4.2	0.28	1.8	0.57	-0.5	0.65
Lt ICA	24.7	0.6	0.6	0.43	3.2	0.41	15.2	0.53	-4.6	0.11	-1	0.94	18.3	0.42	-1.5	0.77
Lt MCA basal	6.7	0.05	2.3	0.08	2.9	0.02	5.4	0.12	-1	0.57	1.5	0.56	4.8	0.12	-0.6	0.48
Lt MCA pial	3.4	0.41	-0.3	0.68	-0.3	0.75	1.1	0.46	-0.5	0.33	-0.4	0.78	0.9	0.50	-0.2	0.53
Lt PCA basal	11.3	0.25	1.6	0.09	1.7	0.19	3.9	0.53	0	0.86	-0.3	0.66	4.6	0.54	-0.5	0.22
Lt PCA pial	0.2	0.87	1.9	0.34	2.3	0.42	-0.1	0.97	-1	0.64	-2.8	0.50	-2	0.59	0.3	0.06
Lt VA	-5.8	0.53	0.5	0.59	1.3	0.29	-6.7	0.3	1.9	0.13	-10.8	0.80	-4	0.9	0.8	0.07
Lt SCA, AICA, PICA	5.2	0.02	2.3	0.03	3.2	0.03	0	0.06	1.1	0.38	-0.7	0.63	2	0.02	0.5	0.75
Rt ACA basal	-6.6	0.43	1.9	0.15	1.5	0.16	-3	0.61	1.2	0.94	3.4	0.22	-3.3	0.71	-0.1	0.28
Rt ACA pial	6.3	0.51	-0.9	0.55	-1	0.56	1.9	0.29	-0.8	0.37	0	0.93	1.1	0.47	-0.1	0.91
Rt ICA	-21.5	0.46	-1.8	0.73	-8.9	0.61	-9.6	0.53	-2.3	0.30	5.8	0.34	-10.1	0.32	-0.3	0.9
Rt MCA basal	3.4	0.45	0.3	0.65	0.8	0.43	1.2	0.59	-0.7	0.90	-3.2	0.16	0.4	0.98	0.3	0.52
Rt MCA pial	6.7	<0.01	1.2	0.05	1.7	0.02	4.3	<0.01	-1	0.04	-0.2	0.79	3.3	<0.01	-0.2	0.29
Rt PCA basal	2	0.12	2.2	0.04	2.5	0.03	0.9	0.15	0	0.53	-0.7	0.38	2	0.08	-0.1	0.47
Rt PCA pial	10	0.97	1.4	0.90	0.9	0.94	3.1	0.83	-0.2	0.42	3.3	0.15	0.2	0.59	0.6	0.07
Rt SCA, AICA, PICA	-1.3	0.79	1.8	0.22	1.3	0.59	-2.5	0.4	-0.4	0.98	-0.1	0.89	-1	0.97	0.1	0.26
Rt VA	-2.9	0.4	1.2	0.31	1.6	0.54	-3.6	0.42	2.1	0.48	-5.8	0.21	-1.9	0.51	0	0.49

BA, basilar artery; Lt, left; ACA, anterior cerebral artery; ICA, internal carotid artery of intracranial portion; MCA, middle cerebral artery; PCA, posterior cerebral artery; VA, vertebral artery; SCA, superior cerebellar artery; AICA, anterior inferior cerebellar artery; PICA, posterior inferior cerebellar artery

*The relative changes of the arterial luminal area and the maximal diameter were calculated by the difference between 6 months and initial divided by the initial value and presented as % change.

†The *P*-values were calculated by the Wilcoxon signed rank tests.

Table S4. Association Between Parameters of Symptomatic Arterial Segment and Non-Symptomatic Arterial Segments

	**% change of symptomatic arterial segment features**					
	**Stenosis grade (%)**		**Remodeling index**		**Wall area index**	
Non-symptomatic arterial segment features	*r*	*P*	*r*	*P*	*r*	*P*
Area, mm^2	0.21	0.12	-0.16	0.27	0.1	0.46
Maximum inscribed sphere radius, mm	0.04	0.73	-0.15	0.31	0.03	0.82
Minimal diameter, mm	0.08	0.52	-0.15	0.27	0.04	0.73
Maximal diameter, mm	0.19	0.16	-0.13	0.36	0.11	0.42
Min-max diameter ratio	-0.2	0.16	0.05	0.66	-0.23	0.1
Curvature	-0.02	0.89	-0.04	0.77	-0.05	0.73
Perimeter, mm	0.2	0.14	-0.09	0.51	0.12	0.37
Luminal circularity	-0.14	0.34	-0.22	0.12	-0.27	0.05
